# Regeneration of the periodontium using enamel matrix derivative in combination with an injectable bone cement

**DOI:** 10.1007/s00784-012-0743-z

**Published:** 2012-05-03

**Authors:** Daniël A. W. Oortgiesen, Gert J. Meijer, Antonius L. J. J. Bronckers, X. Frank Walboomers, John A. Jansen

**Affiliations:** 1 Department of Biomaterials (309), Radboud University Nijmegen Medical Center, Nijmegen, The Netherlands; 2 Department of Periodontology and Implantology, Radboud University Nijmegen Medical Center, Nijmegen, The Netherlands; 3 Department of Oral Cell Biology, Academic Center for Dentistry (ACTA), Universiteit van Amsterdam and Vrije Universiteit, Research Institute MOVE, Amsterdam, The Netherlands

**Keywords:** Animals, Calcium phosphates, Periodontal diseases, Guided tissue regeneration, Periodontal

## Abstract

**Objectives:**

Enamel matrix derivative (EMD) has proven to enhance periodontal regeneration; however, its effect is mainly restricted to the soft periodontal tissues. Therefore, to stimulate not only the soft tissues, but also the hard tissues, in this study EMD is combined with an injectable calcium phosphate cement (CaP; bone graft material). The aim was to evaluate histologically the healing of a macroporous CaP in combination with EMD.

**Materials and methods:**

Intrabony, three-wall periodontal defects (2 × 2 × 1.7 mm) were created mesial of the first upper molar in 15 rats (30 defects). Defects were randomly treated according to one of the three following strategies: EMD, calcium phosphate cement and EMD, or left empty. The animals were killed after 12 weeks, and retrieved samples were processed for histology and histomorphometry.

**Results:**

Empty defects showed a reparative type of healing without periodontal ligament or bone regeneration. As measured with on a histological grading scale for periodontal regeneration, the experimental groups (EMD and CaP/EMD) scored equally, both threefold higher compared with empty defects. However, most bone formation was measured in the CaP/EMD group; addition of CAP to EMD significantly enhanced bone formation with 50 % compared with EMD alone.

**Conclusions:**

Within the limits of this animal study, the adjunctive use of EMD in combination with an injectable cement, although it did not affect epithelial downgrowth, appeared to be a promising treatment modality for regeneration of bone and ligament tissues in the periodontium.

**Clinical relevance:**

The adjunctive use of EMD in combination with an injectable cement appears to be a promising treatment modality for regeneration of the bone and ligament tissues in the periodontium.

## Introduction

Periodontal disease leads to tissue destruction, such as epithelial downgrowth and thereby loss of root cementum, periodontal ligament (PDL), and alveolar bone. Currently, the most predictable treatment is scaling and rootplaning of the root surfaces, possibly in combination with surgery. As a drawback, this treatment results only in arresting inflammation, thus preventing or slowing down further attachment loss. As such, the outcome for this type of treatment is healing with a long junctional epithelium [1]. Ideally, lost root cementum, PDL, and alveolar bone, should be regenerated.

Currently, such regeneration is sometimes partially obtained with the use of enamel matrix derivative (EMD; Emdogain®, Straumann, Basel, Switzerland) or guided tissue regeneration (GTR) [2]. For example, a recent rat study using an intrabony defect showed less gingival recession, a deeper sulcus, and shorter junctional epithelium upon EMD treatment [3]. However, bone formation was minimal in both EMD-treated group and untreated control group. Evidently, regeneration of both hard (bone) as well as soft periodontal tissue needs another approach. In view of this, biodegradable calcium phosphate (CaP) cements have been shown excellent candidates for bone regeneration. Such cements largely consist of alpha tri-calcium phosphate to enable adequate in vivo turn over, especially when the cement is made porous by the incorporation of poly(dl-lactic-*co*-glycolic acid) (PLGA) microspheres [4–8]. So far, most tri-calcium phosphate materials used in clinical periodontal regeneration therapy are applied in particulate form and demonstrated to be encapsulated or to be resorbed without evident periodontal regeneration. In contrast, injectable cement compositions have already been reported with promising histological results for periodontal regeneration [9, 10].

For the current investigation, it was hypothesized that combining the positive properties of EMD and an injectable macroporous CaP will have a synergistic effect on periodontal tissue healing. Thus, CaP together with EMD might stimulate bone formation besides the regeneration of the soft periodontal tissues. Therefore, the aim was to evaluate histologically the healing of a macroporous CaP in combination with EMD in a pre-validated defect model in rat [3].

## Materials and methods

### Enamel matrix derivative

EMD (30 mg/mL) was used according to the manufacturer’s recommendations in the designated experimental groups. The exact composition of EMD is not disclosed and the responsible reactive molecules are not yet identified, but EMD is thought to contain at least amelogenins, which are dissolved in the carrier propylene glycol alginate.

### Calcium phosphate cement with PLGA microspheres

#### Preparation of the PLGA microparticles

PLGA with a 50:50 lactic to glycolic acid copolymer ratio was used (molecular weight 17 ± 1.6 kDa; Purasorb®, Purac, Gorinchem, the Netherlands). PLGA microparticles were prepared by an established double-emulsion-solvent extraction technique ((water-in-oil)-in-water) as previously described [7]. Microparticles were produced by injecting 500 μL demineralized water into a tube containing a solution of 1,400 mg PLGA in 2 mL dichloromethane. This mixture was emulsified (60 s) on a vortex (Genie 2, Scientific Industries, Bohemia, New York, USA). Subsequently, 6.0-mL 0.3 % aqueous poly(vinyl alcohol) (PVA) solution was added and emulsified (60 s) on a vortexer to produce the second emulsion. The mixture was then added to 394 mL PVA solution and 400 mL of 2 % aqueous isopropanol solution with rapid stirring (1 h). Subsequently, the microparticles were allowed to settle (15 min) and the solution was decanted. Then, the microparticles were washed and collected through centrifugation (1,500 rpm, 5 min), lyophilized, and stored under argon at −20 °C until further use. The size distribution of the PLGA microparticles (*n* = 100) was determined by image analysis (Leica Qwin®, Leica Microsystems, Wetzlar, Germany). The morphology of the PLGA microparticles was determined by observation with a scanning electron microscope (JEOL 6400-LINK AN 10000 at 10 kV). The PLGA microspheres were 26 ±8 μm in diameter and had a spherical and smooth appearance. The PLGA/CaP cement showed signs of interconnecting pores. More specific details have been previously reported [7].

#### Preparation of PLGA/CaP cement composite

CaP consisted of 85 % alpha tri-calcium phosphate, 10 % dicalcium phosphate anhydrous, and 5 % precipitated hydroxyapatite.

PLGA microparticles were added to the CaP powder in a PLGA/CaP weight ratio of 20/80. For delivery into the defect, a 2-mL syringe (Sherwood Medical, Den Bosch, the Netherlands) was closed at the tip with a stopper and filled with 500 mg of the PLGA/CaP mixture. Preoperatively, syringes filled with PLGA/CaP powder were sterilized by gamma radiation (25 kGy, Isotron B.V., Ede, the Netherlands). Before surgery, sterile cement liquid (1 % Na_2_HPO_4_; 200 μL) was added to provide injectability. Then, the syringe was placed in a mixing apparatus (Silamat®, Vivadent, Schaan, Lichtenstein) and mixed (15 s). The stopper was removed, and the cement was injected into the defect.

### Animals

Fifteen healthy adult Wistar rats, weighing approximately 350–400 g were used. The Animal Ethical Committee of Radboud University Nijmegen (RU-DEC 2008-091) approved the study protocol. All procedures were in accordance with the national guidelines for the care and use of laboratory animals. The animals were screened for good physical condition and were specific-pathogen free. Food pellets and water were provided ad libitum but were withheld overnight pre-operatively.

### Surgical procedures

**Fig. 1:**
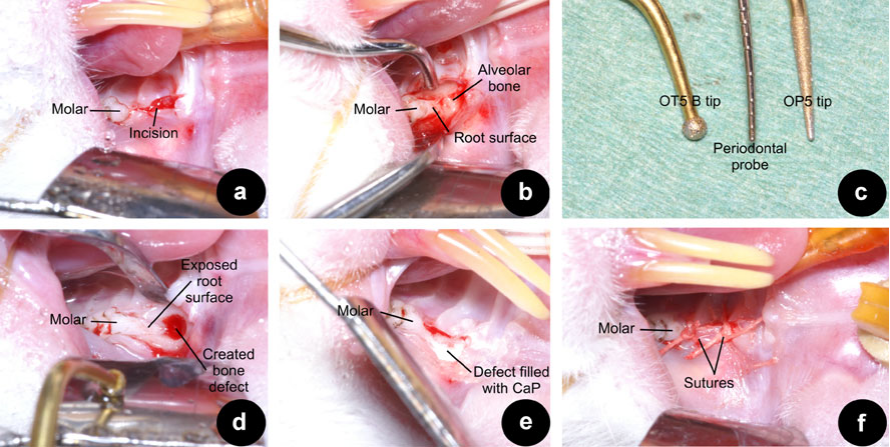
Overview of surgical procedure: **a** a 3-mm incision was made mesial of the maxillary molar; **b** muco-periosteal flaps were raised on the buccal and palatal aspect; **c** the OT5 B tip is displayed next to a standard 10-mm periodontal probe on the *left*, on the *right* observe the OP5-tip; **d** the created defect, prior to the final corrections standardizing the defect; **e** an example of a defect treated with the CaP cement after the application of EMD; **f** the flaps sutured with 4–0 resorbable sutures

**Fig. 2:**
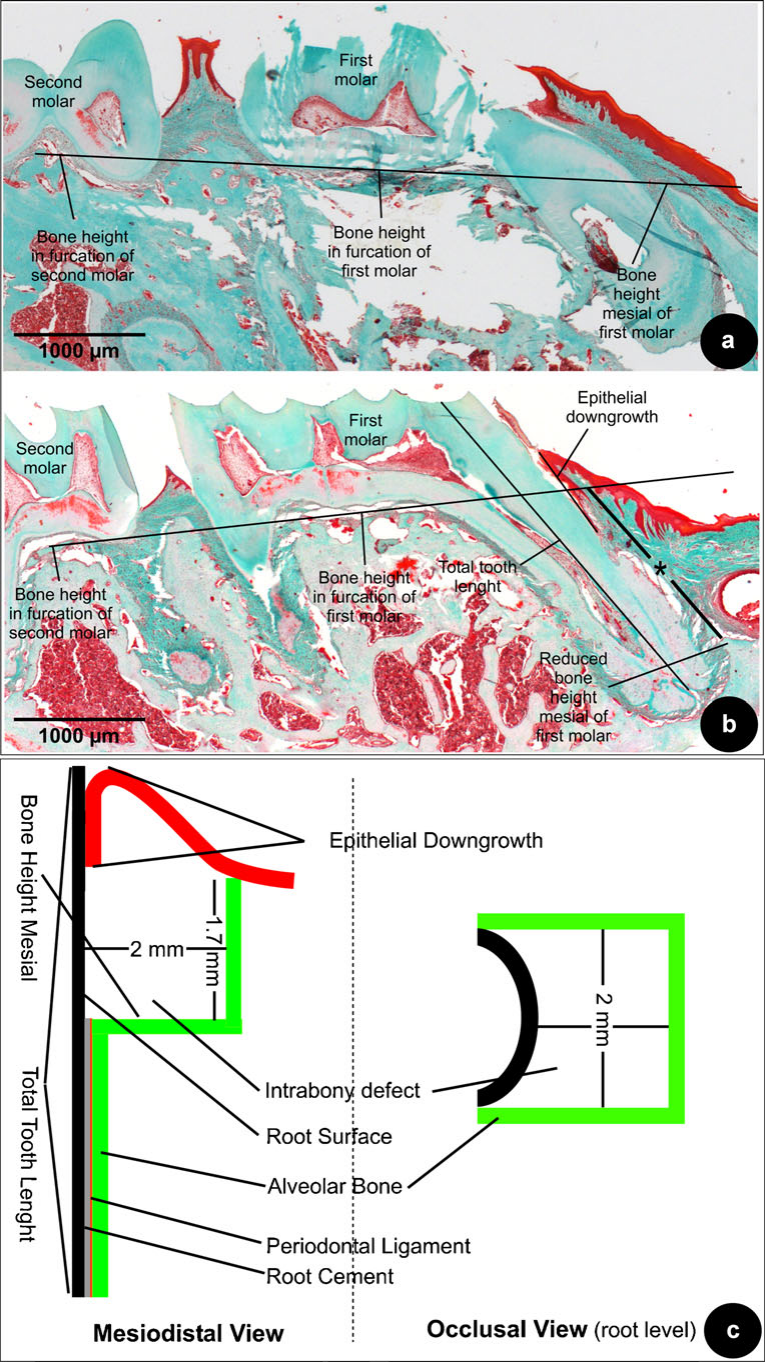
**a** Bone height was measured mesial of the maxillary molar (Masson staining; original magnification, ×2.5). A line connecting the furcations of the neighboring molars was drawn. This *black line* indicates the connected furcations and was then compared with the bone height mesial of the first molar; shown is a non-operated sample indicating that this drawn line indicates the original bone height mesial; **b** the *black line with asterisk* indicates the difference between the original bone height and the reduced bone height after surgery; shown a sample from the EMD-treated group (Masson staining; original magnification, ×2.5); **c** diagram to show the dimensions of the three-wall defect and the measurements taken for the histomorphometrical analysis

Surgery was performed using general inhalation anaesthesia by means of intubation. To minimize peri- and postoperative pain, carprofen (Rimadyl® Pfizer, Capelle aan de IJssel, the Netherlands; 50 mg/mL s.c., 0.01 mL/kg bodyweight) was given pre-operatively, and continued the first 2 days postoperatively. Anaesthesia was maintained by a mixture of nitrous oxide, isoflurane 2–3 %, and oxygen through a constant volume ventilator and monitored, using a pulsoxymeter, to ensure that an appropriate level of anaesthesia was achieved and maintained. During operation, animals were positioned in a head-restraining device to allow proper access to the maxillary molar area. Surgery was performed with the aid of magnifying loupes (×2.5) and strong light by one surgeon (DO). Bilateral intrabony three-wall defects were created mesial of both maxillary first molars. A local, adrenaline containing, dental anaesthetic (Ultracain D-S®, Aventis Pharma BV, Gouda, the Netherlands) was used to reduce the bleeding tendency and for pain management. A 3-mm-long incision was made on the alveolar ridge (Fig. 1a). Flaps were raised in order to expose the tooth and alveolar bone (Fig. 1b). Using a piezoelectric device (OT5 B-tip; 1.7 mm in diameter; Piezosurgery®, Mectron, Carasco, Italy) a three-wall intrabony defect was made along the root surface. Thereafter the residual bone, PDL, and root cementum were carefully removed from the root surface, using a less abrasive tip (OP5-tip) (Fig. 1c). This tip was also used to finalize the size of the defect (width × length × depth; 2 × 2 × 1.7 mm) (Fig. 2c) while constantly carefully monitoring the dimensions using a periodontal probe. Pilot experiments on preparing the defects showed that this method led to very consistent defect sizes as assessed in post-mortem measurements (data not shown). Prior to the designated treatment all root surfaces were treated with 24 % ethylenediaminetetraacetic acid (EDTA; PrefGel™, Straumann®, Basel, Switzerland) during 2 min according manufacturer’s recommendation. Subsequently, the defects were rinsed with sterile saline, dried with a sterile gauze and, if indicated, filled immediately thereafter (Fig. 1d).

Groups were randomly divided over all animals. The first group was left empty for spontaneous healing (controls, *n* = 10). The second group received only EMD (*n* = 10). In the third (CaP/EMD) group first EMD was applied. Subsequently, CaP was injected into the defect (*n* = 10) (Fig. 1e). EMD was used as indicated by the manufacturer. Briefly, after EDTA application the defects were thoroughly rinsed with sterile saline, dried with sterile gauzes and, by pipetting, 15 μL of EMD was applied strictly onto the root surface. After EMD application, a 2-min waiting time for protein adsorption was kept. Subsequently, CaP was used to completely fill the defect to the original contour of the bone, i.e., ~6.8 mm^3^ (2 × 2 × 1.7 = 6.8 mm^3^).

Finally, the flaps were repositioned, using resorbable sutures (Vicryl® 4-0, Ethicon Products, Amersfoort, the Netherlands) (Fig. 1f). During the first ten postoperative days, the animals were fed with powdered food for minimal wound disturbance. After 12 weeks, the animals were euthanized by CO_2_ inhalation, samples were harvested, and processed for histological analysis [3].

### Histological preparation

First, the complete maxilla was dissected and excess tissue removed. After fixation in buffered formaldehyde (pH 7.4) 10 % for 24 h, the specimens were decalcified for 6 h under a low voltage current in a decalcifying bath (TDE™ 30 bath, Sakura Finetek Europa B.V., Zoeterwoude, The Netherlands) and subsequently dehydrated in a graded series of ethanol (70–100 %). Before embedding, the molars were stained with black ink to ensure that sections were made in the area of interest. Maxillas were split in the middle, and the hemi maxillas were embedded in Paraplast paraffin (Klinipath B.V., Duiven, the Netherlands). Sections of 6 μm were cut in mesio-distal direction, stained with hematoxylin–eosin and trichrome (Masson modification Goldner). Light microscopical evaluation of all sections was done using an optical microscope (Leica MZ12, Leica BV, Rijswijk, the Netherlands) and consisted of a complete morphological qualitative description and quantitative analysis of the tissue response. For the quantitative analysis, a histological scoring system was developed and a comprehensive assessment was performed on three sections per specimen for each of both staining in the center of the original defect site [11, 12]. Each section was assigned a score and from the scores of all sections in one group the average score of the groups was calculated. These assessments were separately performed by two examiners blinded to treatment group.

### Histomorphometry and statistical analysis

Following histomorphometrical parameters were assessed:

1. Root damage and root resorption were scored on a 2-point grading scale (yes/no) based upon the histological images. Root damage was recorded when sharp, straight substance removal could be observed, i.e., caused by instrumentation. Root resorption was documented when irregular substance removal could be observed. A contingency table was made and data were compared using a Chi-square test, in a statistical program (Instat version 3.05, GraphPad Software, San Diego, CA, USA).
2. Inflammation was scored on a 4-point scale (Table 1). Subsequently, groups were compared with a two-way analysis of variance (ANOVA) with post-hoc Tukey testing. A higher score represents less inflammation.
3. Epithelial downgrowth was measured from the gingival margin to the most apical extent of the junctional epithelium (Fig. 2b). The total tooth length was measured from the apex to the mesial cusp of the molar. To compensate for deviations in alignment of the histological slides, relative values were used by dividing the length for epithelial downgrowth by the total tooth length. Before analysis, these values were inverted, i.e., a higher score represents a more beneficial tissue response (less epithelial downgrowth). Hereafter, each score received a rank number and comparisons between groups were made using ANOVA and post-hoc Tukey testing.
4. PDL formation and connective tissue attachment (CT) were scored on a 4-point grading scale (Table 1). PDL formation included new (a) cellular cementum formation in combination of perpendicularly oriented fibers inserted into the cement and bone. CT attachment was regarded as the supracrestal connective tissue adhesion to the root surface without new cementum formation but in presence of perpendicularly orientated of fibers [13, 14]. Subsequently, groups were compared with ANOVA and post-hoc Tukey testing. A higher score represents better periodontal healing.
5. The bone height of the defect located mesially of the maxillary molar was measured as follows. First a reference line was drawn interconnecting the furcation heights of the neighboring molars (Fig. 2a; a non-operated sample). Subsequently, from this reference line to the bottom of the created defect, a second line (with asterisk) was drawn parallel to the mesial root of the first molar (Fig. 2b; a sample from the EMD group). Alike epithelial downgrowth, this difference was measured as a relative value to the total tooth length. All scores in each group were sorted low to high, i.e., a higher score indicated more bone height. Finally, each score received a rank number and a comparison between the groups was made using ANOVA and post-hoc Tukey testing.

**Table 1:** Quantitative histological scoring system for inflammation and periodontal healing

Response	Score	Description
Inflammation	1	Masses of inflammatory cells
	2	Many inflammatory cells, showing some fibroblasts
	3	Immature connective tissue, showing fibroblasts with few inflammatory cells
	4	Normal appearance of connective tissue with few inflammatory cells
Periodontal healing	0	No PDL regeneration, no connective tissue adhesion, random supracrestal fiber orientation and, epithelial downgrowth
	1	No PDL regeneration but CT adhesion with perpendicular fiber orientation supracrestally
	2	Evidence of PDL regeneration limited to the apical part of the defect and, CT adhesion with perpendicular fiber orientation supracrestally
	3	Complete PDL regeneration

## Results

### General observations

All surgical and recovery procedures were uneventful except for the rats treated with the CaP; during the first postoperative 2–3 days considerable extra-oral swelling was observed. Still, all animals gained weight in a comparable manner. No other macroscopic signs of inflammation were observed during the entire healing period.

### Descriptive histology

The periodontal defects could still be detected in all samples. In some samples (10 out of 30) the tiny bone wall to the sinus cavity was penetrated. The acellular cementum was always completely removed from the root surface together with the supporting alveolar bone, which only occasionally resulted in a slightly damaged root. In some samples, of different treatment groups, root resorption could be observed except, for the EMD-treated groups.

**Fig. 3:**
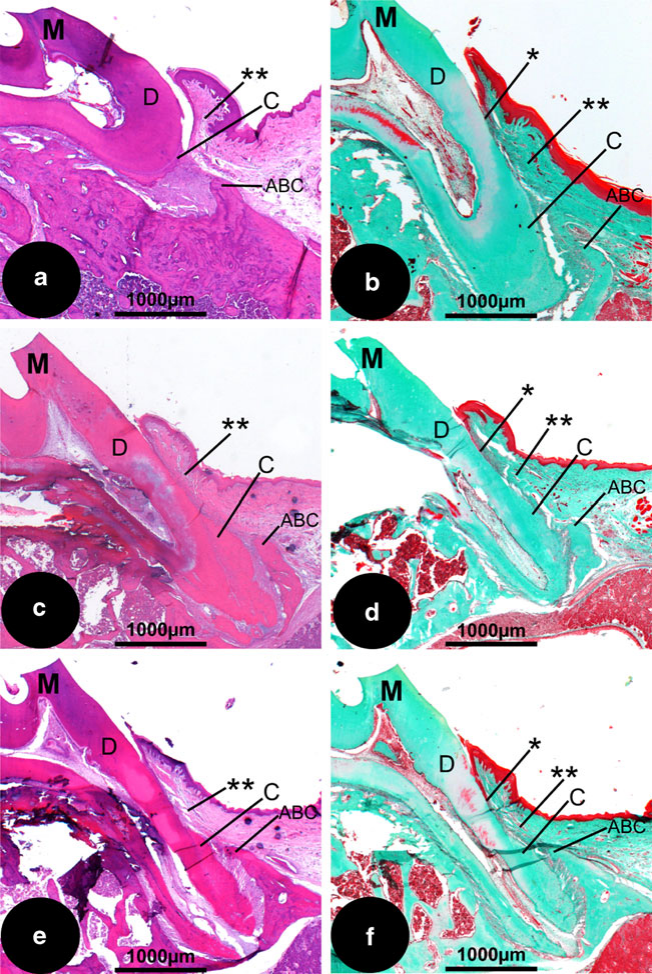
Histological overview of the different treatment groups. **a** Empty defect (HE staining; original magnification, ×2.5); **b** empty defect (Masson staining; original magnification, ×2.5); **c** EMD (HE; original magnification, ×2.5); **d** EMD (Masson; original magnification, ×2.5); **e** CaP/EMD (HE; original magnification, ×2.5); **f** CaP/EMD (Masson; original magnification, ×2.5). *ABC* alveolar bone crest, *C* cementum, *D* dentin of the root, *M* indicating the first molar of the maxilla, projected all in the same fashion (apex down). *Asterisk*, apical extent of the junctional epithelium. *Double asterisk*, supra-alveolar tissues

**Fig. 4:**
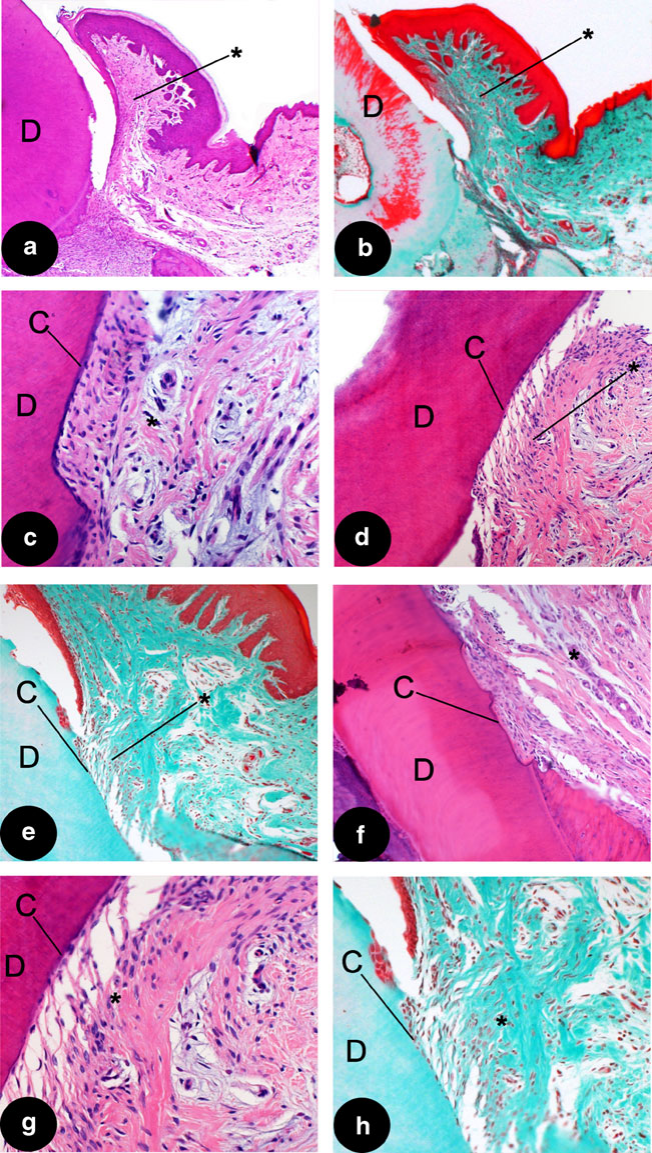
Detail**s** of different treatment groups. **a** Supra-crestal tissues (*asterisk*); showing random orientation of the fibers in the empty group (HE; original magnification, ×2.5), please observe the supra-crestal tissues are detached from the rootsurface, indicating a weak bond between the two tissues; **b** another example of an empty group sample (Masson; original magnification, ×2.5); **c** detail of a functionally orientated supra-crestal connective tissue attachment (*asterisk*), showing new cementum formation (**c**) in a EMD sample (HE; original magnification, ×20); **d** detail of a functionally orientated supra-crestal connective tissue attachment (*asterisk*), showing new cementum formation (**c**) in another EMD sample (HE; original magnification, ×10); **e** detail of a functionally orientated supra-crestal connective tissue attachment, showing new cementum formation in a sample treated with CaP/EMD (Masson; original magnification, ×10); **f** supra-crestal connective tissue attachment in CaP/EMD group (HE; original magnification, ×10); **g** higher magnification of (**d**) showing new cementum (**c**) (HE; original magnification, ×20); **h** higher magnification of (**e**) showing new cementum (**c**) (Masson; original magnification, ×20). *C* cementum, *D* dentin of the root. *Asterisk*, supra-alveolar tissues

A common finding in the empty group was epithelial downgrowth to the level to which the cementum was removed (Fig. 3a, b). In addition, empty defects showed very limited signs of new cementum, new bone or PDL regeneration. A collapse of the soft tissues could be observed as a consequence of the bony defect. A detailed image of the supra-crestal tissue is presented in Fig. 4a, b. Consistently, in all sections a random orientation of the supra-crestal fibers instead of the normal structured perpendicular orientation was observed.

Within the EMD-treated group limited evidence of bone formation was seen (Fig. 3c, d). Some samples (3 out of 10) showed evidence of cementum formation with PDL fibers embedded into the pre-existing bone, which was limited to the apical part of the defect and always in proximity of pre-existing cementum (Fig. 4c, d). Overall, a very limited inflammatory response was observed in all EMD specimens. The supra-crestal connective tissues showed functional orientation of the fibers (Fig. 4c, d). In the specimens that had not formed new cementum, always a CT adhesion was present.

The CaP/EMD group (Fig. 3e, f) showed for PDL and CT comparable histological results with the EMD group; i.e. supra-crestal, functionally orientated fibers (Fig. 4e, f). In the CaP/EMD group consistently more bone formation was observed in the apical part of the defect compared with the empty and EMD groups. A few samples (two out of ten) showed remnants of the CaP, however, never in contact with the root surface.

### Histomorphometry and statistical analysis

In all groups damage and resorption of the roots, inflammatory response, epithelial downgrowth, healing of periodontal fibrous tissue and bone healing were measured. In each assessment, a higher score was indicative for a more favorable tissue response. As previously mentioned, all data were collected by two examiners that were blinded to the treatment performed. The inter-examiner agreement was already initially very high. However in case of discrepancy in score between the two examiners, this was dissolved by a combined viewing of the specific specimen and subsequent discussion always led to an agreement on the score to be used for the specimen.

In all groups some root damage was observed; three samples out of the empty group (*n* = 10), five samples out of the EMD group (*n* = 10), and six out of the CaP/EMD group (*n* = 10). This was also the case for root resorption, four samples out of the empty group (*n* = 10), two samples out of the CaP/EMD group (*n* = 10), however the EMD group did not exhibit root resorption. There were no statistically significant differences in the amount of root damage and resorption between all groups (*p* = 0.89 and 0.89 respectively), indicating that root damage or resorption were no confounding parameters of influence in the analysis of any group.

**Fig. 5:**
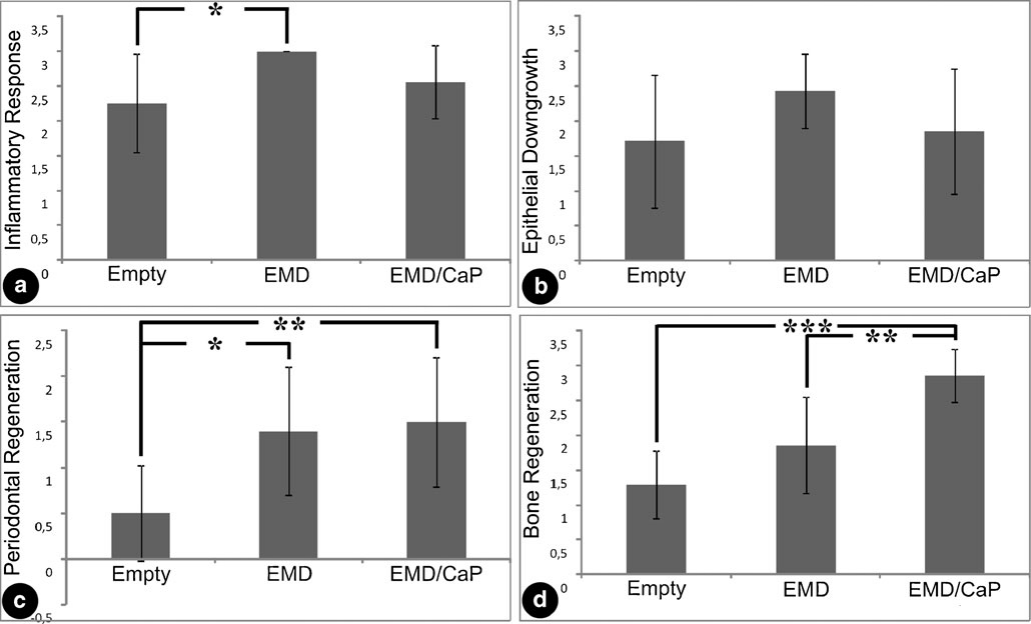
Histomorphometrical data. **a** Inflammatory response, **b** epithelial downgrowth, **c** periodontal regeneration, and **d** bone regeneration. *P < 0.05; **P < 0.01; ***P < 0.001

Least inflammation was noted in the EMD group and most in the empty group (Fig. 5a) (*p* < 0.05). The group treated with EMD and CaP, showed an average degree of inflammation, in between that of both other groups.

The least epithelial downgrowth was measured in the EMD-treated group. The results for the other groups were quite similar, and not significantly different from the EMD group (Fig. 5b).

For PDL and CT healing, the empty group showed the worst score whereas both experimental groups (EMD or CaP/EMD) performed significantly better, increasing threefold from 0.5 (empty) to 1.4–1.5 (EMD or CaP/EMD) (Fig. 5c).

Bone was least formed in the empty group, while most bone was formed in the CaP/EMD group. (Fig. 5d). Inclusion of CAP to EMD significantly enhanced bone formation in the EMD group, from score 1.9 to 2.9; an increase of approximately 50 %.

## Discussion

The best treatment of periodontal defects, today, is only to stop the destructive process that is called periodontitis. If at all possible, the periodontal condition should be brought back into its original condition. For this purpose, regenerative techniques come into view. As in periodontal defects both soft tissues (PDL and CT) as also hard tissues (cement and alveolar bone) need to be reconstructed, we hypothesized that the combination of EMD with a calcium phosphate cement (CaP) could accomplish such periodontal healing. To date, defects left empty, showed merely repair. EMD application, indeed showed periodontal soft tissue (PDL and CT) healing, however, limited bone regeneration was observed. The combination of EMD and CaP appeared to have a synergistic effect, stimulating both soft periodontal tissue healing and bone regeneration.

Regarding our animal model, it is obvious that the herein used model has certain limitations. Canine models, as regularly used in periodontal regenerative studies, allow for example preparation of larger defects. Also a notch in the root, that can serve as a reference point for future histological evaluation, can be prepared. Obviously, the model used for this study is too small for such a notch, however in the near future perhaps in vivo (micro-)CT might be feasible to allow for more accurate, even three-dimensional, measurements.

In the present study small three-wall defects were created artificially in rats. It might be assumed that this size of defect will heal spontaneously and does not require the use of biomaterials. However, as can be observed in our results, the empty three-wall defects in fact showed limited spontaneous healing. We feel that in spite of the overall dimensions, the defect size is still relatively large defect to this small animal model.

Another point of debate in the field of preclinical periodontal research is whether defects should be created surgically, like in this study, or induced chronically (i.e., plaque infection), or even the combination of both. Positive aspects of plaque-induced periodontal breakdown are that a diseased root surface is created. However, also this situation is still not completely similar to natural occurring periodontitis. An even more important point is that defects may vary between animals, making subsequent treatment and analysis challenging or even impossible. Additional disadvantages are prolonged experimental time due to additional handling of the animals. Surgical creation of defects on the other hand has certain positive aspects, such as that defect sites can be standardized and creation is much faster. The animal model used in this study does not fully represent a periodontal disease state and likewise has several limitations as a model for periodontal regeneration (e.g., differences in periodontal tissues and histopathological features). Nevertheless, the currently used model is cost-effective and especially suitable for initial studies.

Regarding our study setup, the handling of the cement needs further attention. Although it appeared possible to create defects of the same size, the bone architecture in the defect region varied from one rat to another. Due to this variation, occasionally, the bone separating the sinus mucosa from the periodontal defect was damaged, which resulted in bleeding, thereby frustrating to get the defect dry prior to applying EMD and/or CaP. Moisture compromises the setting of the CaP, and as a consequence, might have reduced the mechanical strength of the CaP as also was reported for a rat cranial defect [15]. Another aspect is that sometimes residue of CaP material could be observed. In order to obtain fast resorption of the grafts, a low molecular PLGA was included in the CaP cement. However, after 12 weeks of healing still some grafted CaP was seen, which corroborates with other studies in which CaP was injected in periodontal [16] or other bone defects [6].

In the CaP-treated group, all rats showed initial swelling, which has not been reported before [6, 16, 18]. It is has been described that graft particles, in the region of interaction between the graft and host bone, can cause an inflammatory response [19]. In our study, due to possible initial instability of the CaP, such fragments might have caused this transient inflammatory response and associated swelling.

Another pit fall is primary loss of CaP after its application. To date, in a human study, the authors mentioned that in 6 out of the 15 patients treated with CaP, such an exfoliation did occur [9]. Although in the present study flaps could always be closed without compromise, as also the animals were monitored for graft exfoliation during the first postoperative days and in addition powdered food was offered, exfoliation can never be excluded totally [10, 17]. In a recent human study using a particulated biphasic calcium phosphate (BCP) either or not with EMD, graft exfoliation was not described; this might be due to differences in the used grafting materials; particulated versus injectable that needs to set in situ. However, clinically, no advantages were reported when BCP was used in combination with EMD [20].

No differences in epithelial down growth could be observed for the combined therapy with CaP and EMD or EMD alone in our study. This is exactly in line with what others found in clinical research. For example, a recent meta-analysis also showed no benefits for combination therapies with EMD [21]. In this meta-analysis, numerous different materials and EMD have been compared. In this meta-analysis, clinical data have been compared but no histological data have been reported. Even though in the clinic the difference in clinical parameters might not justify combined use, this might be different on a histological level. Therefore, we feel that combination therapy is worth further histological evaluation for potential benefits for clinical treatment.

Most periodontal regeneration studies are conducted in dogs, which allows careful plaque control, the application of local antiseptics and even brushing. This is impossible in rats, which might have affected the results negatively. From human GTR studies, it is well known that plaque control is of upmost importance [22].

As bone remodeling is different between humans, rats, and canines, graft-material-associated changes may not be as apparent in the human situation where there is a lower rate of remodeling [23].

The histological and histomorphometrical findings indicate that the reparative type of healing, as observed in the empty group in our experiment, is consistent with numerous published reports [1, 3, 13, 24]. Our experiment showed that even three-wall periodontal defects did not regenerate spontaneously. Although these empty defects were also treated with EDTA, like both experimental groups, it is well know from literature that EDTA treatment does not have any benefits as mono-therapy [25, 26]. Consistent with literature, ankylosis near the CaP cement was never observed [16, 27]. One study reports formation of cementum and PDL between the root and CaP [16]. The authors postulated that CaP acts much like a “membrane” in providing space maintenance and wound stabilization. Possibly, in our study the CaP cement was initially not stable enough to elucidate similar effects.

Using the same rat model, comparable results were found for empty defects (no regeneration), or when EMD was used (regeneration of the PDL but no bone formation) [3]. Also in a dog study, empty defects showed only a reparative type of healing while EMD-treated defects demonstrated varying amounts of new cementum but hardly any regenerated bone volume [13]. Adding CaP, however, with or without EMD showed significant more cementum and bone regeneration. In this study, the importance of soft tissue support was emphasized while avoiding immediate contact between the biomaterial and the root [13]. Follow-up investigations have to be confirmed whether CaP cement only serves as wound stabilizer or if its osteoconductive properties are the determining factor.

## Conclusions

Within the limits of this rat study, the application of EMD, either or not, in combination with calcium phosphate cement enhanced PDL formation. The additional use of an injectable calcium phosphate cement resulted in more bone formation compared with an empty defect or a defect treated with EMD only. The combination of EMD and calcium phosphate cement resulted in the most favorable effects for respectively PDL formation and bone regeneration. Although no effect on epithelial down growth could be assessed, the adjunctive use of calcium phosphate in combination with EMD is a promising treatment modality for regeneration of bone and ligament tissues in the periodontium.

## References

[1] Caton J, Nyman S, Zander H (1980). Histometric evaluation of periodontal surgery. II. Connective tissue attachment levels after four regenerative procedures. J Clin Periodontol.

[2] American Academy of Periodontology (2005). Research, Science and Therapy Committee. Periodontal regeneration. J Periodontol.

[3] Nemcovsky CE, Zahavi S, Moses O, Kebudi E, Artzi Z, Beny L, Weinreb M (2006). Effect of enamel matrix protein derivative on healing of surgical supra-infrabony periodontal defects in the rat molar: a histomorphometric study. J Periodontol.

[4] Link DP, van den Dolder J, van den Beucken JJ, Cuijpers VM, Wolke JG, Mikos AG, Jansen JA (2008). Evaluation of the biocompatibility of calcium phosphate cement/PLGA microparticle composites. J Biomed Mater Res A.

[5] Link DP, van den Dolder J, van den Beucken JJ, Habraken W, Soede A, Boerman OC, Mikos AG, Jansen JA (2009). Evaluation of an orthotopically implanted calcium phosphate cement containing gelatin microparticles. J Biomed Mater Res A.

[6] Link DP, van den Dolder J, van den Beucken JJ, Wolke JG, Mikos AG, Jansen JA (2008). Bone response and mechanical strength of rabbit femoral defects filled with injectable CaP cements containing TGF-beta 1 loaded gelatin microparticles. Biomaterials.

[7] Habraken WJ, Wolke JG, Mikos AG, Jansen JA (2006). Injectable PLGA microsphere/calcium phosphate cements: physical properties and degradation characteristics. J Biomater Sci Polym Ed.

[8] Habraken WJ, Wolke JG, Mikos AG, Jansen JA (2008). PLGA microsphere/calcium phosphate cement composites for tissue engineering: in vitro release and degradation characteristics. J Biomater Sci Polym Ed.

[9] Shirakata Y, Setoguchi T, Machigashira M, Matsuyama T, Furuichi Y, Hasegawa K, Yoshimoto T, Izumi Y (2008). Comparison of injectable calcium phosphate bone cement grafting and open flap debridement in periodontal intrabony defects: a randomized clinical trial. J Periodontol.

[10] Sorensen RG, Wikesjö UM, Kinoshita A, Wozney JM (2004). Periodontal repair in dogs: evaluation of a bioresorbable calcium phosphate cement (Ceredex) as a carrier for rhBMP-2. J Clin Periodontol.

[11] Jansen JA, Dhert WJ, van der Waerden JP, von Recum AF (1994). Semi-quantitative and qualitative histologic analysis method for the evaluation of implant biocompatibility. J Invest Surg.

[12] Jansen JA, de Ruijter JE, Janssen PT, Paquay YG (1995). Histological evaluation of a biodegradable polyactive/hydroxyapatite membrane. Biomaterials.

[13] Shirakata Y, Yoshimoto T, Goto H, Yonamine Y, Kadomatsu H, Miyamoto M, Nakamura T, Hayashi C, Izumi Y (2007). Favorable periodontal healing of 1-wall infrabony defects after application of calcium phosphate cement wall alone or in combination with enamel matrix derivative: a pilot study with canine mandibles. J Periodontol.

[14] Blumenthal NM, Koh-Kunst G, Alves ME, Miranda D, Sorensen RG, Wozney JM, Wikesjö UM (2002). Effect of surgical implantation of recombinant human bone morphogenetic protein-2 in a bioabsorbable collagen sponge or calcium phosphate putty carrier in intrabony periodontal defects in the baboon. J Periodontol.

[15] Plachokova A, Link D, van den Dolder J, van den Beucken J, Jansen J (2007). Bone regenerative properties of injectable PGLA-CaP composite with TGF-beta1 in a rat augmentation model. J Tissue Eng Regen Med.

[16] Hayashi C, Kinoshita A, Oda S, Mizutani K, Shirakata Y, Ishikawa I (2006). Injectable calcium phosphate bone cement provides favorable space and a scaffold for periodontal regeneration in dogs. J Periodontol.

[17] Xu HH, Takagi S, Sun L, Hussain L, Chow LC, Guthrie WF, Yen JH (2006). Development of a nonrigid, durable calcium phosphate cement for use in periodontal bone repair. J Am Dent Assoc.

[18] Sugawara A, Fujikawa K, Takagi S, Chow LC (2008). Histological analysis of calcium phosphate bone grafts for surgically created periodontal bone defects in dogs. Dent Mater J.

[19] Shanbhag AS, Macaulay W, Stefanovic-Racic M, Rubash HE (1998). Nitric oxide release by macrophages in response to particulate wear debris. J Biomed Mater Res.

[20] Pietruska M, Pietruski J, Nagy K, Brecx M, Arweiler NB, Sculean A (2012) Four-year results following treatment of intrabony periodontal defects with an enamel matrix derivative alone or combined with a biphasic calcium phosphate. Clin Oral Investig (in press) 10.1007/s00784-011-0611-2 21881869

[21] Tu YK, Woolston A, Faggion CM (2010). Do bone grafts or barrier membranes provide additional treatment effects for infrabony lesions treated with enamel matrix derivatives? A network meta-analysis of randomized-controlled trials. J Clin Periodontol.

[22] Cortellini P, Pini-Prato G, Tonetti M (1994). Periodontal regeneration of human infrabony defects (V). Effect of oral hygiene on long-term stability. J Clin Periodontol.

[23] Bloebaum RD, Ota DT, Skedros JG, Mantas JP (1993). Comparison of human and canine external femoral morphologies in the context of total hip replacement. J Biomed Mater Res.

[24] Sculean A, Berakdar M, Willershausen B, Arweiler NB, Becker J, Schwarz F (2006). Effect of EDTA root conditioning on the healing of intrabony defects treated with an enamel matrix protein derivative. J Periodontol.

[25] Yamamoto S, Masuda H, Shibukawa Y, Yamada S (2007). Combination of bovine-derived xenografts and enamel matrix derivative in the treatment of intrabony periodontal defects in dogs. Int J Periodontics Restorative Dent.

[26] Parashis AO, Tsiklakis K, Tatakis DN (2006). EDTA gel root conditioning: lack of effect on clinical and radiographic outcomes of intrabony defect treatment with enamel matrix derivative. J Periodontol.

[27] Shirakata Y, Oda S, Kinoshita A, Kikuchi S, Tsuchioka H, Ishikawa I (2002). Histocompatible healing of periodontal defects after application of an injectable calcium phosphate bone cement. A preliminary study in dogs. J Periodontol.

